# Inflammatory disease status and response to TNF blockade are associated with mechanisms of endotoxin tolerance

**DOI:** 10.1016/j.jaut.2024.103300

**Published:** 2024-08-07

**Authors:** Felix IL. Clanchy, Federica Borghese, Jonas Bystrom, Attila Balog, Henry Penn, Dobrina N. Hull, Rizgar A. Mageed, Peter C. Taylor, Richard O. Williams

**Affiliations:** a Kennedy Institute of Rheumatology, Nuffield Department of Orthopaedics, Rheumatology and Musculoskeletal Sciences, University of Oxford, Roosevelt Drive, Oxford, United Kingdom; b Centre for Cancer Cell and Molecular Biology, Barts Cancer Institute, Queen Mary University of London, London, United Kingdom; c Department of Rheumatology and Immunology, Szent-Györgyi Albert Clinical Centre, University of Szeged, Szeged, Hungary; d Northwick Park Hospital, Harrow, United Kingdom; e Centre for Translational Medicine and Therapeutics, William Harvey Research Institute, Queen Mary University of London, London, United Kingdom; f Botnar Research Centre, Nuffield Department of Orthopaedics, Rheumatology and Musculoskeletal Sciences, University of Oxford, Oxford, United Kingdom

**Keywords:** Endotoxin, monocytes, Rheumatoid arthritis, Ankylosing spondylitis, Inflammation

## Abstract

The mechanisms of endotoxin tolerance (ET), which down-regulate inflammation, are well described in response to exogenous toll-like receptor ligands, but few studies have focused on ET-associated mechanisms in inflammatory disease. As blocking TNF can attenuate the development of ET, the effect of anti-TNF on the expression of key ET-associated molecules in inflammatory auto-immune disease was measured; changes in inflammatory gene expression were confirmed using an ET bioassay. The expression of immunomodulatory molecules was measured in a murine model of arthritis treated with anti-TNF and the expression of ET-associated molecules was measured in whole blood in rheumatoid arthritis (RA) and ankylosing spondylitis (AS) patients, before and after therapy. The expression of ET-associated genes was also measured in RA patient monocytes before and after therapy, in anti-TNF responders and non-responders. *Tnfaip3, Ptpn6* and *Irak3* were differentially expressed in affected paws, spleens, lymph nodes and circulating leucocytes in experimental murine arthritis treated with anti-TNF. Prior to therapy, the expression of *TNFAIP3, INPP5D, PTPN6, CD38* and *SIGIRR* in whole blood differed between human healthy controls and RA or AS patients. In blood monocytes from RA patients, the expression of *TNFAIP3* was significantly reduced by anti-TNF therapy in non-responders. Prior to therapy, anti-TNF non-responders had higher expression of *TNFAIP3* and *SLPI*, compared to responders. Although the expression of *TNFAIP3* was significantly higher in RA non-responders prior to treatment, the post-treatment reduction to a level similar to responders did not coincide with a clinical response to therapy.

## 1 Introduction

Inflammation is a common physiological process that eliminates pathogens or repairs tissue damage. Chronic inflammation may arise from several causes, including auto-immune disease. A failure to return to the steady state may be caused by inadequate immuno-modulatory mechanisms such as regulatory cells (Tregs, Bregs, “myeloid suppressor” cells), regulatory mediators (IL-10, sTNFR, ILRa) or signalling molecules (SOCS, STAT3). A failure of immunomodulatory mechanisms may occur at many points in the resolution of inflammation, potentiating the development of chronic inflammation.

Endotoxin tolerance (ET) is a regulatory mechanism that results in the refractory phenotype observed in macrophage (Mϕ)-lineage cells after sustained or repeated stimulation with Toll-like receptor (TLR) ligands; TNF signaling facilitates the establishment of ET [1,2]. Various SNPs in genes that contribute to the ET phenotype are associated with inflammatory autoimmune disease. Thus, susceptibility to several inflammatory diseases is influenced by polymorphisms of *TNFAIP3* (reviewed in Mele, A et al. [3]) and the development of asthma has been demonstrated to be associated with variants of *IRAK3* [4,5]. Similarly, a variant of *PTPN6* was observed to be associated with conditions as diverse as IBD [6] and inflammatory skin conditions [7]; in mice, deficiency in the orthologous gene is associated with the murine *motheaten* phenotype [8]. Several other genes (*INPP5D* [9–11], *SIGIRR* [12,13], *CD38* [14], *CXCL10* [15] and *SLPI* [16]) have been identified as bio-markers of ET or implicated in ET-associated processes, and therefore, if dysregulated, may potentiate inflammatory disease.

As damage-associated molecular patterns are present in chronically inflamed tissues, and can induce the expression of inflammatory mediators via TLRs, ET-associated molecules may be upregulated and promote the resolution of inflammation; however, endogenous or exogenous influences may override or attenuate their activity. The development of ET can be prevented in vitro by cytokines such as interferon-γ [17] (IFNγ) or by blocking the action of tumour necrosis factor (TNF) [18]. TNF blockade (e.g. etanercept, infliximab, adalimumab) may therefore affect the upregulation of ET-associated molecules in patients.

Various studies have explored the broad aspects of TNF blockade in inflammatory disease but few have focused on multiple mechanisms of ET, using multiple autoimmune diseases and models. As these conditions and models are amenable to TNF blockade and, given that TNF has a significant role in the establishment of ET, the relationship of ET-associated molecules and disease severity or treatment response was further examined.

In this study, we assessed the expression of key ET-associated molecules in a murine model of arthritis treated with anti-TNF and in human auto-immune disease – rheumatoid arthritis (RA) and ankylosing spondylitis (AS) - treated with anti-TNF. We sought to determine the relationship between ET-associated biomarkers and disease status in murine experimental arthritis and human auto-immune inflammatory disease. Specifically, the stage of disease, the response to anti-TNF and differences between anti-TNF responders and non-responders were assessed with reference to biomarker expression. Additionally, we determined the correlation of inflammatory and ET biomarkers with a canonical ET bioassay.

## 2 Methods and materials

### 2.1 Human clinical samples

Blood and tissues were collected in accordance with approval from the Riverside Research Ethics Committee, the City and East London Ethics Committee, the Ethics Committee of the Ministry of Health of Hungary and Ethics Committee of the University of Szeged; poor response to therapy was defined as a reduction in DAS28 of less than 2.2. Clinical blood samples were obtained from the Nuffield Orthopaedic Centre (Oxford), The Royal Free London NHS Foundation Trust (London), Northwick Park Hospital (London), Imperial College Healthcare Trust hospitals (London) and the Department of Rheumatology and Immunology, University of Szeged after receiving informed consent.

#### 2.1.1 Human whole blood

Blood was obtained from patients prior to and after 3 months of anti-TNF therapy. Blood from RA patients, AS patients and healthy controls was collected in Paxgene Blood RNA tubes (PreAnalytiX) and then stored at –80 °C until mRNA was extracted using the Paxgene Blood RNA kit (Qiagen) according to the manufacturer’s instructions.

#### 2.1.2 Human RA blood monocytes

Blood for monocyte isolation was obtained from RA patients prescribed anti-TNF, including adalimumab and etanercept, prior to and after 3 months of treatment as previously described [19]. Briefly, PBMC were isolated by density gradient separation. Monocytes were isolated by CD14 positive selection (Miltenyi). Purity was confirmed by flow cytometric staining of CD14 (HCD14) and CD16 (3G8) which were obtained from BioLegend Ltd. An Isolate II RNA/DNA/Protein kit (Bioline) was used to extract mRNA. Reverse transcription was performed as mentioned below under *RT-PCR* and gene expression was measured with Taqman qPCR assays as detailed in the Supplementary methods, using premixed qPCR mastermix (Eurogentec) and a ViiA7 Real Time PCR system thermocycler (ThermoFisher Scientific).

### 2.2 Monocyte-derived macrophages

Monocyte-derived Mϕ were differentiated from healthy control monocytes isolated from the blood of normal donors (NHS Blood Service). Monocytes were cultured for 5 days in 10 % FBS RPMI (10^7^ cells/10mL/10 cm dish) supplemented with 50 ng/mL M-CSF (Peprotech) as previously described [20]; Mϕ were detached and re-plated into 6 well plates, allowed to attach for 24h, then stimulated as described under *ET bioassay*.

### 2.3 ET bioassay

**Fig. 1:**
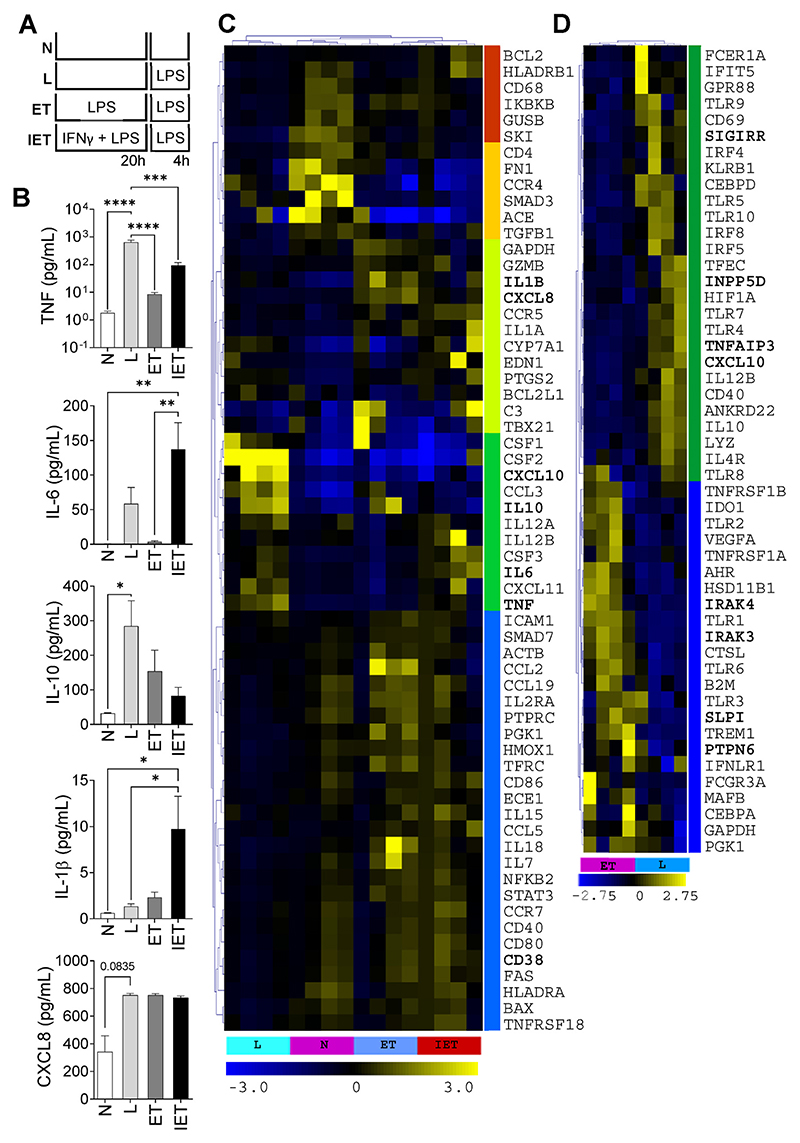
A human monocyte-derived Mϕ endotoxin tolerance bioassay. Inflammatory mediators measured by immuno-assay with 4 conditions (A) comprising a 20 h pretreatment, removal of media and rinsing of cells followed by a stimulation of 4 h; Nil (N) no treatment, LPS 4 h with no pretreatment (L), induction of endotoxin tolerance (ET) by low dose LPS for 20 h followed by LPS 4 h, IFNγ-attenuated ET (IET) – as with ET but with IFNγ during pretreatment. (B) Cytokine secretion TNF, IL-6, CXCL8 (IL-8), IL-1β and IL-10; *p < 0.05, **p < 0.01, ***p < 0.001, ****p < 0.0001, ANOVA, n = 4 donors. (C) Heat map and hierarchical clustering of common inflammatory gene expression for ET bioassay conditions (see Supplementary Fig. 1 for further particulars). (D) Heat map and hierarchical clustering of myeloid gene expression comparing L and ET (see Supplementary Fig. 2 for further particulars).

To compare Mϕ phenotypes, a bioassay comprised of four conditions was optimized based upon previous research [17,21]. In addition to (a) unstimulated cells, 3 conditions were used – (b) no pre-treatment and 4 h of LPS stimulation (10 ng/mL), (c) the endotoxin tolerant state was induced by pre-treating for 20 h with LPS 1 ng/mL with a second stimulation for 4 h with LPS 10 ng/mL and lastly (d) the effect of IFNγ was demonstrated by pretreating cells with LPS (1 ng/mL) and IFNγ (40 ng/mL, Peprotech) for 20hrs and then stimulating for 4 h with LPS 10 ng/mL (see Fig. 1A). After pre-treatment, the cells were washed twice with PBS prior to stimulation. After the 4-h stimulation, the supernatant was collected to assess secreted mediators by immunoassay (TNF, IL-1β, IL-6, IL-10, CXCL8 (Mesoscale Discovery)) and the cells were lysed for the extraction of RNA which was reverse transcribed to cDNA for qPCR analysis.

### 2.4 Collagen-induced arthritis

All procedures were approved by the Animal Welfare Ethical Review Board and were undertaken in accordance with project and personal licences issued by the UK Home Office under the UK Animals (Scientific Procedures) Act, 1986. DBA/1J male mice (8–10 weeks old) were immunized as previously described [20]. Briefly, from ~14 days from the immunization, the mice developed paw swelling and a clinical score ranging from 0 to 3 was assigned to each paw according to the presence of inflammation; naïve mice were used as controls to compare to ET-associated genes expressed in the physiological steady state.

At ten days after the onset of disease, the mice were humanely euthanized and the paws, lymph nodes, spleens were harvested and blood was obtained via cardiac puncture; where specified, cells were extracted from CIA joints and stained for CD115 expression (AFS98, BioLegend Ltd) flow cytometric analysis, as previously described [22]. Blood was collected into heparin-coated tubes and erythrocytes were lysed to obtain leucocytes. Paws were snap-frozen in liquid nitrogen and pulverized with the BioPulverizer™ (BioSpec). Paw powder was then homogenised in 500 μL of TRIzol reagent (Invitrogen) using the Sample Grinding Kit (GE Healthcare). The aqueous phase of the phenol/-chloroform extraction was mixed with 70% ethanol then added to an RNA extraction column (RNeasy Mini Kit, Qiagen), and RNA extraction was completed according to manufacturer’s instructions. Spleens and lymph nodes were pressed through a cell strainer, washed, and then RNA was extracted using Trizol in a similar fashion to the paws.

### 2.5 RT-PCR

Reverse transcription of 500 ng of RNA was performed with the High Capacity cDNA Reverse Transcription Kit (Applied Biosystems) in a 40 μL reaction which was then diluted to a total of 120 μL. Expression of the target genes was determined by TaqMan gene expression assays (ThermoFischer Scientific) and expressed relative to housekeeper gene expression using the δδCT approximation method; see Supplementary Methods for Taqman qPCR assay details.

### 2.6 Statistical analysis

Data was analysed by ANOVA or Student’s t-test as detailed in the figure legends, using Prism (GraphPad). Heatmaps were created using Multi-Experiment Viewer (TM4 Software) [23].

## 3 Results

### 3.1 Endotoxin tolerance bio-assay

In Mϕ, prolonged stimulation with a TLR ligand leads to a refractory state unless the inflammatory milieu contains mediators which augment the pro-inflammatory Mϕ phenotype. By LPS re-stimulation of Mϕ in a classical ET bioassay (Fig. 1A) we confirmed previous findings, such as reduced production of TNF, IL-6 and IL-10, but not other cytokines such as IL-1β or CXCL8 (Fig. 1B). In addition, we demonstrated that ET is not merely a down-regulation of gene expression or an exhausted phenotype (as evidenced by, for example, continued secretion of IL-1β and CXCL8) but rather a reprogramming of gene expression, as evidenced by Fig. 1C wherein inflammatory gene expression clusters into five broadly defined groups. A comparison of the expression of myeloid genes in naïve and ET-conditioned Mϕ stimulated with LPS (Fig. 1D) indicates that pre-treatment with a low dose TLR-ligand elicits a different response to the same stimulation; see Supplementary Figs. 1 and 2 for further particulars of Fig. 1(C and D). A general reduction in the expression of M1 markers was observed in the ET stimulation which, broadly speaking, acquired a phenotype similar to M2-polarised Mϕ. In the IET treatment, the tolerizing action of 20 h pre-treatment with LPS was abolished by the presence of IFNγ, as previously described [24]. Of particular interest were the gene profiles of the cytokines measured in Fig. 1B, which correlated with secreted cytokine profiles.

### 3.2 Collagen-induced arthritis

We next addressed the question of whether ET-associated mechanisms are active in a murine model of chronic inflammatory disease (collagen-induced arthritis) and whether they contribute to disease resistance and/or remission. Thus, the expression of immunomodulatory genes was measured at different stages of disease and compared between joints that developed disease versus those that did not. The time points day 5 and 10 in the pre-onset period were chosen to assess the gene expression at the beginning and toward the end of the T cell expansion and migration phase. Affected paws were harvested at day 1 (early arthritis) and 10 (late phase) of the inflammatory process. Unaffected paws were collected at day 10 to investigate the effect of inflammatory mediators derived from arthritic lesions via the blood, in joints lacking clinical signs of inflammation [19].

**Fig. 2:**
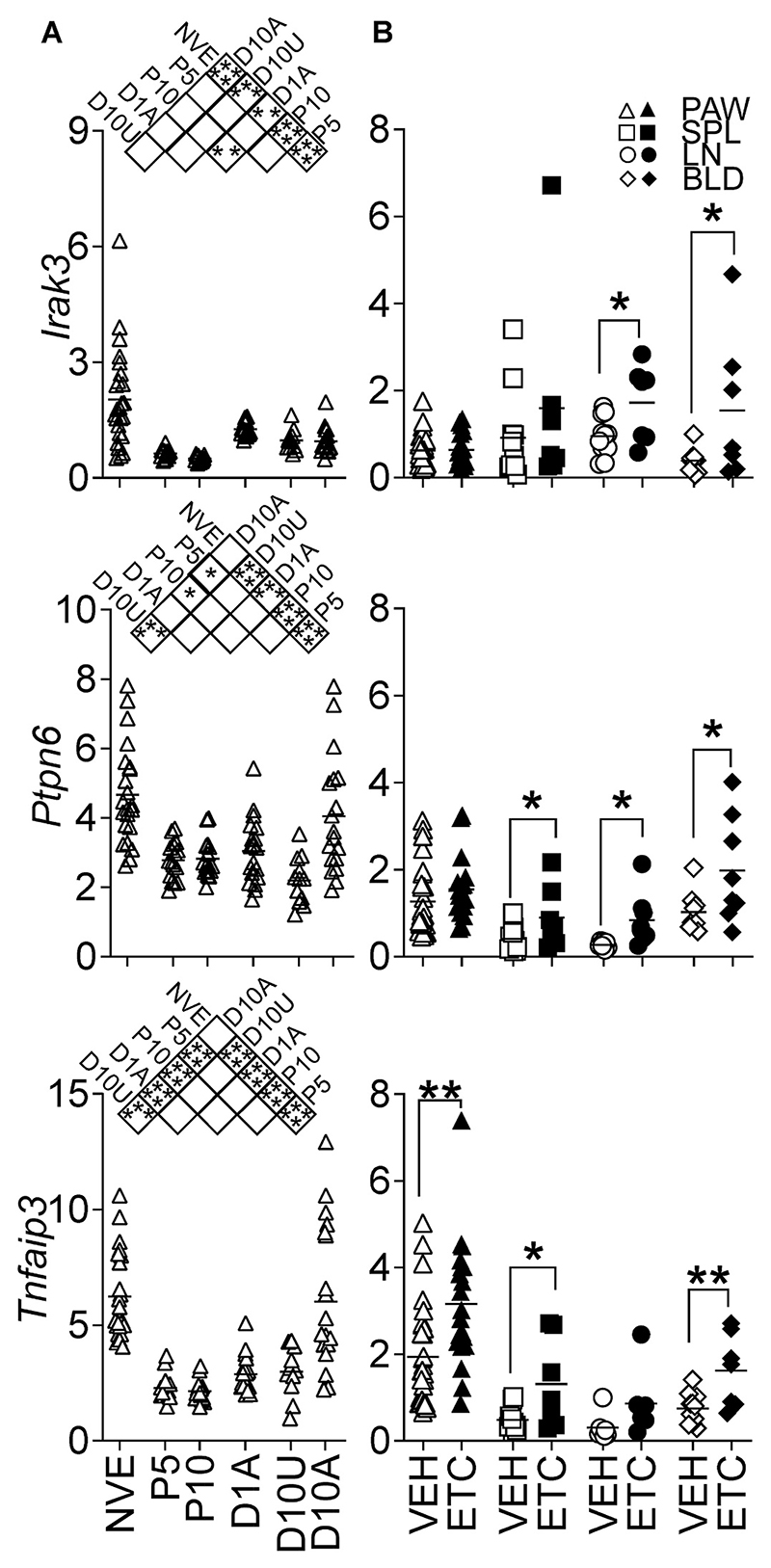
Immunoregulatory gene expression in CIA. (A) The expression of *Irak3, Ptpn6* and *Tnfaip3* was measured in mice at different stages of disease in CIA – Naïve, un-immunized mice (NVE), immunized mice prior to disease onset and 5 or 10 days after immunization (P5 and P10 respectively), affected paws from mice on the first day of arthritis (D1A) and from day 10 post onset unaffected and affected paws (D10U and D10A, respectively); values are mean ± SEM, *p < 0.05, **p < 0.01***p < 0.001, ****p < 0.0001, ANOVA, n = 12–22. (B) ET-associated gene expression in arthritic paws, spleens, lymph nodes (LN) and erythrocyte-lysed blood leucocytes was measured 10 days after disease onset in mice treated with vehicle (VEH) or etanercept (ETC); *p < 0.05, **p < 0.01, *t*-test.

Three key genes were assessed for changes. *I*rak3 is a key ET-associated molecule which attenuates TLR and IL-1β signalling and is largely restricted to Mϕ-lineage cells in humans; the expression has also been reported in other cell types [25]. *T*nfaip*3* is also associated with ET and is expressed haematopoietic and non-haematopoietic cells [26]. *P*tpn*6* is a phosphatase with many binding partners and is most highly expressed in haematopoietic cells where it blocks multiple inflammatory signalling pathways [27]. As shown in Fig. 2A, the kinetics of *Tnfaip3* and *Ptpn6* expression in joints differed from that of *Irak3* in untreated CIA. While *Tnfaip3* and *Ptpn6* levels at the beginning and at the end of the inflammatory process were similar, *Irak3* was reduced soon after immunization, remaining stably lower than the naïve group up to at least 10 days post-onset.

As ET is a phenotype of Mϕ-lineage cells, the proportion of these cells in the joints of mice with differing disease status was measured by flow cytometry expression of the Mϕ-lineage marker CD115 (M-CSF receptor). As anticipated, affected paws from arthritic mice had a higher proportion of CD115^+^ cells after 10 days of disease, compared with the unaffected paws from the same mice (Sup. Fig. 3A and B) or paws harvested at earlier time-points. *Tnf* gene expression was also found to be upregulated in affected paws (Sup. Fig. 3C). These results were some-what unexpected as they show lower levels of *Irak3* in an environment enriched with CD115^+^ cells and with relatively higher levels of *Tnf*, a potent inducer of *Irak3*.

As a central mediator of inflammation, TNF has been demonstrated to modulate not only the inflammatory process but also immunoregulatory processes, including ET [28]. TNF blockade was found to increase the expression of several key immunomodulatory genes (notably *Tnfaip3*) in the arthritic paws and in leucocytes, in comparison to vehicle-treated mice (Fig. 2B) after 10 days of treatment with etanercept.

### 3.3 Human autoimmune disease

As the changes in ET-associated genes in experimental arthritis were associated with disease status and response to therapy, we measured a broad range of ET-associated genes in peripheral blood cells of patients treated with TNF blockade to identify clinically relevant disease markers. In addition to *TNFAIP3, PTPN6* and *IRAK3* measured in murine arthritis, other ET-associated genes were measured (*INPP5D, SIGIRR, IRAK4, CD38, CXCL10* and *SLPI*) in the leucocytes of patients treated with anti-TNF for AS and RA. *INPP5D* expression may be decreased by an allele that raises the risk of Alzheimer’s disease [11], and has been identified as influencing disease processes in MS [10] and helminth infection [9]; the expression of *INPP5D* is increased in ET [29]. Certain *SIGIRR* polymorphisms have been observed to alter susceptibility to TB [12], and are more frequent in RA [13]. IRAK4 is a central TLR/IL-1β signaling molecule to which IRAK3 binds [30]. The expression of CD38 in monocytes increases after prolonged stimulation with LPS and is associated with an exhausted phenotype [14]. CXCL10 production is significantly higher in monocytes in which ET has been induced [15]. Mice deficient for *SLPI* have substantially greater susceptibility to endotoxin shock, but similar levels of serum TNF in response to LPS [16].

### 3.4 ET-associated gene profiles in whole blood

**Fig. 3:**
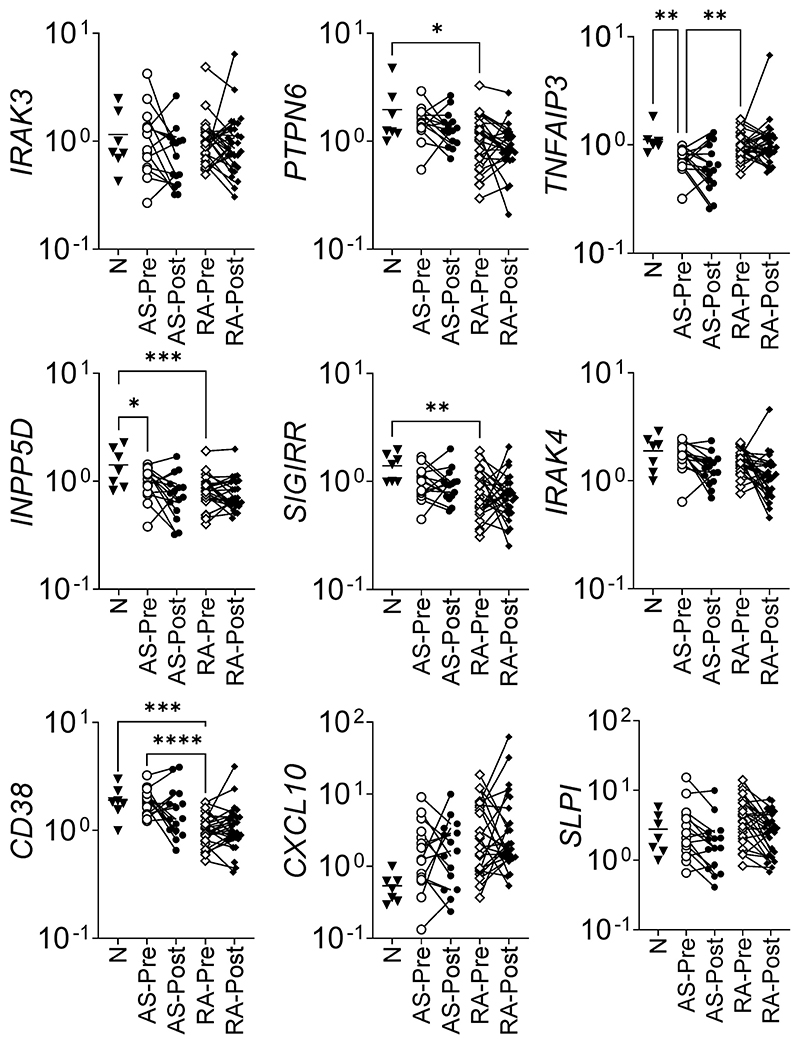
Whole blood expression of ET-associated genes in AS and RA. Whole blood gene expression of ET-associated genes pre- and post-treatment with TNF blockade in ankylosing spondylitis (AS, n = 15) and rheumatoid arthritis (RA, n = 28). A comparison was made with healthy normal controls (N, n = 7); *p < 0.05, **p < 0.01, ***p < 0.001, ****p < 0.0001 ANOVA.

The expression of several genes associated with regulation of inflammatory signalling was measured in the whole blood of patients before and after anti-TNF treatment, and in normal, healthy controls (Fig. 3). When comparing healthy controls, pre-treatment AS and pre-treatment RA patients, we observed reduced *TNFAIP3* expression in AS patients compared to healthy controls and RA patients. RA patients prior to treatment also had reduced expression of *PTPN6, CD38* and *SIGIRR* compared to healthy controls; healthy controls had higher expression of *INPP5D* compared to pre-treatment patient groups. No significant differences were observed between pre and post treatment samples although there was a trend towards declining expression of *IRAK4*, with which IRAK3 interacts, in AS and RA patients by a pairwise comparison (AS p = 0.003, RA p = 0.071, *t*-test).

### 3.5 ET-associated gene profiles in monocytes

**Fig. 4:**
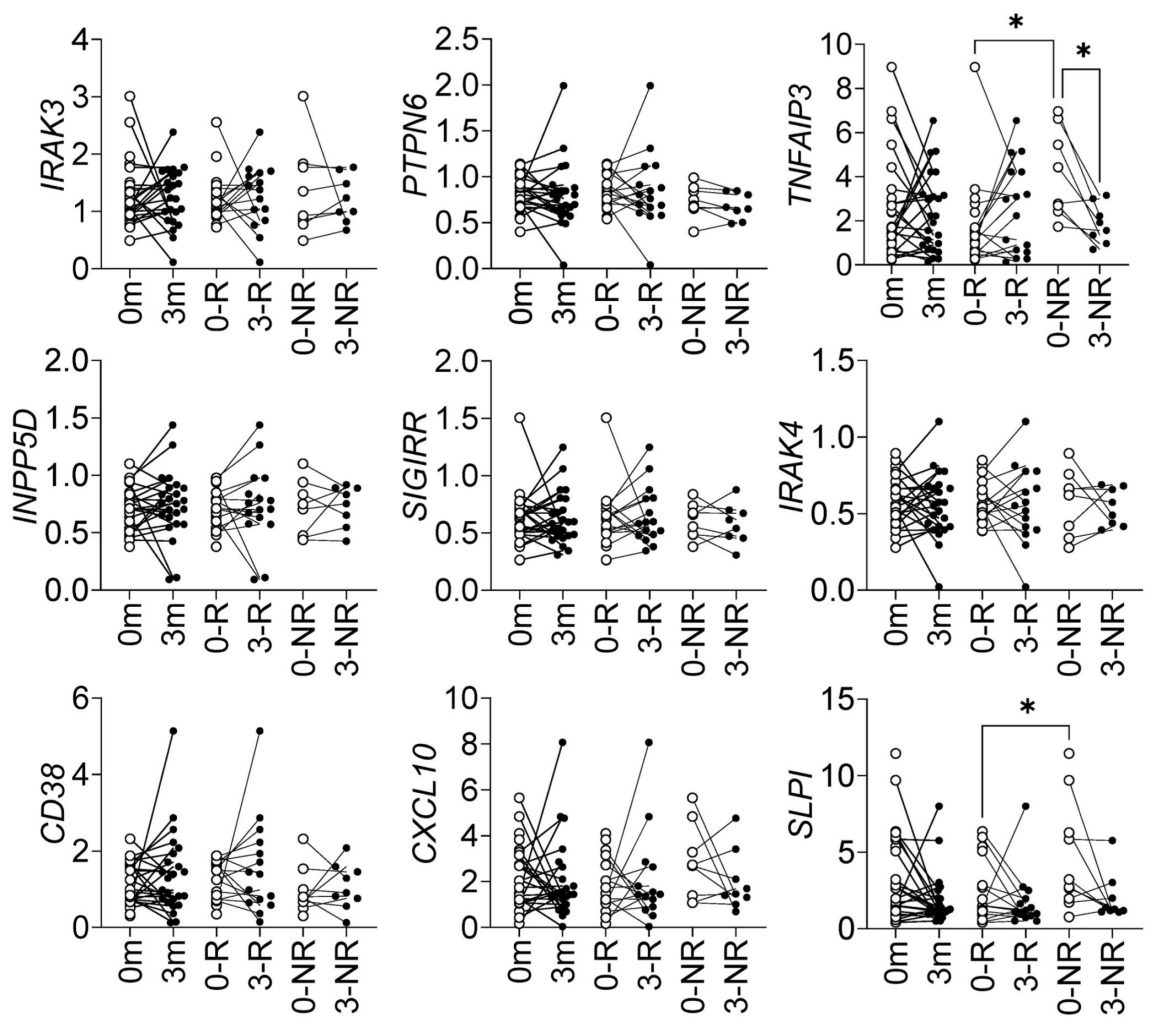
Expression of ET-associated genes in RA monocytes. ET-associated gene expression in monocytes from RA patients (n = 28) before (0m, open symbols) and after (3m, filled symbols) anti-TNF therapy in the entire cohort, and then divided into responders (0-R and 3-R) and non-responders (0-NR and 3-NR); *p < 0.05 ANOVA.

The measurement of gene expression in purified leucocyte subsets permits the delineation of phenotypic changes which may be obscured by an assessment of mixed cells or heterogeneous tissues. To further characterize the mechanisms of ET in response to TNF blockade in RA, monocytes were purified prior to and after treatment, and key genes were measured by qPCR (Fig. 4). In non-responders (NR), *TNFAIP3* was significantly reduced by anti-TNF treatment. Interestingly, there was a significant difference between responders (R) and NR *TNFAIP3* and *SLPI* expression prior to treatment, with NR having higher expression.

## 4 Discussion

When measuring the differential expression of ET-associated genes in vivo, we observed that inflammation in joints was not uniformly associated with increased expression of ET-associated genes, despite the abundant presence of Mϕ and expression of TNF. Nevertheless, when treated with anti-TNF, there were consistent changes in ET-associated genes in CIA. Analysis of clinical samples from patients treated with anti-TNF also demonstrated differential expression of ET-associated genes, the most consistent of which was TNFAIP3.

### 4.1 ET bioassay

Several researchers have proposed in vitro assays to better characterize ET and have demonstrated the potential of TNF to induce ET, and for inflammatory mediators such as IFNγ to prevent ET induction [24, 31,32]. Based on these studies, our ET bioassay had 4 conditions – no stimulation (N), L (LPS 4h), ET (LPS 20h, LPS 4h) and IET (IFNγ/LPS 20h, LPS 4h) and produced inflammatory cytokine responses concordant with prior studies. A comparison of inflammatory genes expressed in the L and ET conditions demonstrated a coordinated reduction in M1 gene expression in the ET condition, which was changed by pretreatment with IFNγ, as previously described [24].

With the exception of *TLR1, 2* and *6* (which form heterodimers upon activation), the TLRs appeared to be expressed at a lower level in the ET Mϕ compared with the L Mϕ. In particular, *TLR4* expression was downregulated in the ET condition, as previously described in other models of ET [33,34]. There was relatively lower expression of *IRF5* and 8 in ET Mϕ, and an increase in the gene expression of *MAFB*. IRF5, which promotes the expression of classical M1 genes such *IL12B, IL23A, IL1β, TNF, CCL3, CCL5, CD40, CD86* and *CCR7* [35], was expressed at higher levels in the L condition, as was *IRF8*, which is also associated with M1 polarization [36]. IRF4 has been described as an M2 Mϕ-associated transcription factor [37] but it was expressed in human ET Mϕ at lower levels compared with the LPS stimulation alone while MAFB, a M2-associated transcription factor, was expressed at higher level in ET Mϕ [38].

ET Mϕ had increased expression of some canonical ET-associated markers such as *IRAK3, PTPN6* and *AHR* [39,40] while other genes usually associated with ET (*TNFAIP3, INPP5D, SIGIRR*), were expressed at lower levels at the level of gene expression in ET-Mϕ compared to L-stimulated Mϕ at the time-points used in this assay. Interestingly, higher gene expression of *TREM1* was found in ET-stimulated Mϕ comparing with the L stimulated cells. TREM1 is a pro-inflammatory receptor associated with a M1 polarization [41] but previous studies have also described the induction of TREM1 by LPS stimulation [42] and an increase of TREM1 gene expression levels in ET [43]. The effect of IFNγ on ET was observed at the gene level for *CCR7, CD38, CSF3, CXCL10, CXCL11, IL6, IL12B* and *TNF*. Conversely, a trend towards increased expression of *C3, IL7, IL18, HMOX1, NFKB2, PTGS2* and *PTPRC* in the ET condition and also the IET condition (compared to LPS alone), suggests a subset of genes are increased in ET and less sensitive to the effect of IFNγ in this assay.

### 4.2 TNF blockade

TNF is a cytokine which induces an inflammatory cascade of other mediators and has been validated as a therapeutic target in various forms of arthritis (rheumatoid, juvenile, psoriatic), AS, ulcerative colititis and Crohn’s disease [44]. The immuno-regulatory effects of TNF are less well characterized, although TNF pretreatment has been shown to be protective in several models of inflammation such as arthritis [45] and EAE [46]. In Mϕ, TNF selectively inhibits IL12 production in an IL-10-dependent manner [47,48]. In mice lacking TNF, inflammatory arthritis was exacerbated, with greater numbers of T cells and higher IFNγ production [49]. The administration of anti-TNF exacerbates disease in MS [50] and EAE [51]. RA patients treated with anti-TNF have a paradoxical increase in IL23 expression and prevalence of Th17, and murine models of arthritis display similar effects [52–54]. TNF facilitates the development of ET by upregulating the expression of key ET-associated molecules, especially TNFAIP3 [1] and IRAK3 [2]; mice lacking these genes exhibit enhanced inflammatory responses and exhibit higher lethality in response to LPS [55,56]. As etanercept is an effective treatment for CIA [57], the higher expression of immunomodulatory genes is congruent with a lack of inflammation; however, specifically blocking a key inducer of *Irak3* and *Tnfaip3* might have been anticipated to reduce their expression.

### 4.3 Comparison of murine and human ET-associated responses

TNF blockade-treated mice have upregulated expression of ET-associated genes compared to vehicle-treated mice. This difference is as much a product of different disease status as a direct effect on individual cells however, the systemic increase of immunomodulatory genes is consistent across the tissues and genes assessed. As CIA is effectively treated by etanercept, the model has some similarity with RA anti-TNF Responders. In comparison, when measuring differences pre and post treatment in human whole blood samples, there were fewer differences, although pre-treatment AS patients had lower *TNFAIP3* compared to normal healthy patients and RA patients prior to treatment. A consistent trend in RA patients prior to treatment compared to healthy donors was the reduced expression of *PTPN6, INPP5D* and *SIGIRR*; this tendency was not observed for *IRAK3* or *TNFAIP3*.

*Tnfaip3* and two other profiled genes show lower expression at P5 and P10 compared to naïve mice paws. Although mouse joints in immunized mice (yet to develop observable disease) may not directly compare to purified Mϕ populations in culture, the consistent reduction in ET-associated gene expression after immunization suggests a link to imminent loss of self-tolerance. Notably, there is a measurable increase in expression for the three genes (statistically significant for *Irak3*) in affected paws on the day of disease onset. Increased *TNFAIP3* expression was observed with concurrently increased TNF expression; in the bioassay comparing L vs ET conditions and murine day 10 affected vs unaffected paws, TNF expression is higher in the former (L and D10Aff), and *TNFAIP3* expression follows the same trend. This is consistent with *TNFAIP3* being a TNF-induced gene. In purified monocytes from RA patients, the greatest differences were observed in the expression of *TNFAIP3*, firstly in differential expression between R and NR prior to therapy, and secondly as observed in the NR patients, who had comparable expression to R after TNF-blocking therapy without a sustained reduction in disease activity. It is of interest that a biomarker might distinguish between R and NR prior to treatment and yet the loss of differential expression after treatment does not result in a similar disease status.

The influence of endogenous factors on ET is an active area of research and the development of liquid biopsies for patients that incorporate IFNγ or TNF inhibitors into a canonical ET assay may be informative for the potential to modulate disease status by different treatments. The effect of TNF and TNF inhibition on the induction of ET appears to be dependent on the assay and cell types used. As some researchers have reported that TNF blockade had limited effect in preventing ET induction [58,59], while others have demonstrated a role for TNF in ET induction and that TNF induces the expression of ET-associated molecules [18,60–64], further research is required for the development of assays for use in a clinical setting. Extrapolation of findings from mice to man requires caution due to species differences as well as the relatively acute nature of the CIA model. Nevertheless, inclusion of this model allowed for time-course studies which would not have been possible with human clinical samples. An additional limitation of both the human and murine analyses is that the analysis of gene expression from heterogeneous cells may reflect changes in cell composition as well as changes in phenotype; both changes are likely to be indicative of changes in disease status.

## 5 Conclusions

In this study we measured a change in the gene expression of immunomodulatory molecules (*Irak3, Ptpn6, Tnfaip3*) in a murine model of RA throughout disease and when treated with TNF blockade; an expanded panel of immunomodulatory genes (*IRAK3, PTPN6, TNFAIP3, INPP5D, SIGIRR, CD38, CXCL10, IRAK4* and *SLPI*) was assessed in clinical samples from AS and RA patients. In whole blood from pre-treatment AS patients, *TNFAIP3* was significantly lower compared to normal controls and RA pre-treatment samples, suggesting suboptimal expression of this gene may be a contributory factor in the pathogenesis of AS; *CD38* also differed between RA and AS patients prior to treatment. Increased *TNFAIP3* was a feature of NR RA monocytes prior to therapy, and was significantly reduced by treatment in the same group; *SLPI* was also higher in monocytes from NR prior to treatment. Further studies are required to elucidate the pathophysiological significance of these findings, and further refine the clinical relevance of the effect of blocking TNF-induced regulatory mechanisms, such as ET.

## Supplementary Material

Supplementary Material Supplementary data to this article can be found online at https://doi.org/10.1016/j.jaut.2024.103300.
