# Supplementary material for: Inflammatory disease status and response to TNF blockade are associated with mechanisms of endotoxin tolerance
Source: J Autoimmun. Author manuscript; Available in PMC 2026 Mar 28. (PMC7618948; doi:10.1016/j.jaut.2024.103300)
Supplement: Supplementary Material — Supplementary data to this article can be found online at https://doi.org/10.1016/j.jaut.2024.103300. [file EMS212790-supplement-Supplementary_Material.zip › 1-s2.0-S0896841124001343-mmc4.pdf]

Supplementary Methods

Human

Gene	Assay
ACE	Hs00174179_m1
ACTB	Hs99999903_m1
AHR	Hs00169233_m1
ANKRD22	Hs00944018_m1
B2M	Hs99999907_m1
BAX	Hs00180269_m1
BCL2	Hs00153350_m1
BCL2L1	Hs00169141_m1
C3	Hs00163811_m1
CCL19	Hs00171149_m1
CCL2	Hs00234140_m1
CCL3	Hs00234142_m1
CCL5	Hs00982282_m1
CCR4	Hs99999919_m1
CCR5	Hs00152917_m1
CCR7	Hs00171054_m1
CD38	Hs01120071_m1
CD4	Hs00181217_m1
CD40	Hs01002915_g1
CD68	Hs00154355_m1
CD69	Hs00934033_m1
CD80	Hs00175478_m1
CD86	Hs00199349_m1
CEBPA	Hs00269972_s1
CEBPD	Hs00270931_s1
CSF1	Hs00174164_m1
CSF2	Hs00171266_m1
CSF3	Hs00357085_g1
CTSL	Hs00964650_m1
CXCL10	Hs00171042_m1
CXCL11	Hs00171138_m1
CYP7A1	Hs00167982_m1
ECE1	Hs00154837_m1
EDN1	Hs00174961_m1
FAS	Hs00163653_m1
FCER1A	Hs00758600_m1
FCGR3A	Hs04188274_m1
FN1	Hs00365052_m1
GAPDH	Hs99999905_m1
GPR88	Hs03027832_s1
GUSB	Hs99999908_m1
GZMB	Hs00188051_m1
HIF1A	Hs00153153_m1
HLA-DRA	Hs00219575_m1
HLA-DRB1	Hs99999917_m1
HMOX1	Hs00157965_m1
HSD11B1	Hs01547870_m1
ICAM1	Hs00164932_m1
IDO1	Hs00984148_m1
IFIT5	Hs01849362_s1
IFNLR1	Hs00417120_m1

Gene	Assay
IKBKB	Hs00395088_m1
IL10	Hs00174086_m1
IL12A	Hs00168405_m1
IL12B	Hs00233688_m1
IL15	Hs00174106_m1
IL18	Hs00155517_m1
IL1A	Hs00174092_m1
IL1B	Hs00174097_m1
IL2RA	Hs00166229_m1
IL4R	Hs00965056_m1
IL6	Hs00174131_m1
IL7	Hs00174202_m1
IL8	Hs00174103_m1
INPP5D	Hs00183290_m1
IRAK3	Hs00936103_m1
IRAK4	Hs00211610_m1
KLRB1	Hs00174469_m1
LYZ	Hs00426232_m1
MAFB	Hs00534343_s1
NFKB2	Hs00174517_m1
PGK1	Hs99999906_m1
PTGS2	Hs00153133_m1
PTPN6	Hs00169359_m1
PTPRC	Hs00365634_g1
SIGIRR	Hs00222347_m1
SKI	Hs00161707_m1
SLPI	Hs00268204_m1
SMAD3	Hs00232219_m1
SMAD7	Hs00178696_m1
STAT3	Hs00234174_m1
TBX21	Hs00203436_m1
TFEC	Hs00992838_m1
TFRC	Hs99999911_m1
TGFB1	Hs00998133_m1
TLR1	Hs00413978_m1
TLR10	Hs01935337_s1
TLR2	Hs01872448_s1
TLR3	Hs01551078_m1
TLR4	Hs00152939_m1
TLR5	Hs01920773_s1
TLR6	Hs01039989_s1
TLR7	Hs01933259_s1
TLR8	Hs00152972_m1
TLR9	Hs00370913_s1
TNF	Hs00174128_m1
TNFAIP3	Hs00234713_m1
TNFRSF18	Hs00188346_m1
TNFRSF1A	Hs00533560_m1
TNFRSF1B	Hs00961749_m1
TREM1	Hs00218624_m1
VEGFA	Hs00900055_m1

Mouse

Gene	Assay
Irak3	Mm00518541_m1
Ptpn6	Mm00469153_m1
Tnfaip3	Mm00437121_m1
Tnf	Mm99999068_m1
Hprt1	Mm03024075_m1
