# Supplementary material for: Inflammatory disease status and response to TNF blockade are associated with mechanisms of endotoxin tolerance
Source: J Autoimmun. Author manuscript; Available in PMC 2026 Mar 28. (PMC7618948; doi:10.1016/j.jaut.2024.103300)
Supplement: Supplementary Material — Supplementary data to this article can be found online at https://doi.org/10.1016/j.jaut.2024.103300. [file EMS212790-supplement-Supplementary_Material.zip › 1-s2.0-S0896841124001343-mmc1.docx]

Sup. Figure 1
